# Restoring Bioenergetics in the Traumatized Eye: The Emergence of Mitochondrial Implantation as a Treatment for Acute Ocular Injury

**DOI:** 10.3390/ijms27219837

**Published:** 2026-11-03

**Authors:** Emma Peveri, Daniella Beiner, Andrew J. W. Huang, Jian-Xing Ma, Amish Asthana, Anthony Atala, Giuseppe Orlando, Yuanyuan Zhang

**Affiliations:** 1 Wake Forest Institute for Regenerative Medicine, Wake Forest University School of Medicine, Winston-Salem, NC 27101, USAamish.asthana@advocatehealth.org (A.A.);; 2 Wake Forest University, Winston-Salem, NC 27109, USA; 3 Department of Ophthalmology and Visual Sciences, Washington University in St. Louis (WashU), St. Louis, MO 63110, USA; 4 Department of Biochemistry, Wake Forest University School of Medicine, Winston-Salem, NC 27101, USA; 5 Translational Eye and Vision Research (TrEVR) Center, Wake Forest University School of Medicine, Winston-Salem, NC 27101, USA; 6 Department of Surgery, Atrium Wake Forest Baptist Medical Center, Wake Forest University School of Medicine, Winston Salem, NC 27101, USA

**Keywords:** mitochondrial transplantation, acute ocular injury, bioenergetic failure, metabolic rescue, ocular trauma

## Abstract

Acute ocular injuries—such as chemical burns, ischemic insults, and mechanical trauma—frequently lead to irreversible vision loss by triggering rapid intracellular adenosine triphosphate (ATP) depletion, mitochondrial calcium overload, severe oxidative stress, and profound mitochondrial dysfunction. Current clinical management primarily focuses on suppressing inflammation and preventing secondary infection, but it fails to address the underlying bioenergetic collapse that drives cellular degeneration. While stem cell and extracellular vesicle therapies have been widely investigated, direct mitochondrial transplantation has recently emerged as a novel strategy for metabolic rescue. This review evaluates the therapeutic potential of exogenously administered mitochondria to mitigate mitochondrial dysfunction and acute trauma in the eye. Emerging preclinical studies demonstrate that transplanted mitochondria can be internalized by selected ocular cell types, conferring short-term cytoprotection, preserving retinal ganglion cell viability, and accelerating corneal epithelial repair. However, robust evidence regarding long-term mitochondrial integration, functional fusion, durable metabolic rescue, and clinical efficacy remains limited. Despite critical challenges in delivery, stability, and dosing, mitochondrial transplantation represents a promising frontier in regenerative ophthalmology that directly targets bioenergetic failure to preserve vision.

## 1. Introduction

### 1.1. Literature Search Strategy

To ensure a comprehensive overview of mitochondrial transplantation in acute ocular injury, a structured literature search was conducted across PubMed/MEDLINE, Embase, Web of Science, and Scopus. Literature published up to July 2026 was evaluated, with the final search executed on 1 August 2026.

Searches were conducted using combinations of Medical Subject Headings (MeSH) and free-text terms:

(“Mitochondrial transplantation” OR “mitochondrial transfer” OR “exogenous mitochondria” OR “mitochondrial delivery”) AND (“ocular injury” OR “corneal burn” OR “retinal ischemia” OR “traumatic optic neuropathy” OR “optic nerve crush” OR “cornea” OR “retina”). Broader secondary searches included keywords such as (“Mesenchymal stem cells” OR “Urine-derived stem cells”) AND (“mitochondrial transfer” OR “bioenergetics”).

Inclusion Criteria: (1) Peer-reviewed original research studies (in vitro, in vivo animal models, or clinical reports) investigating therapeutic mitochondrial transfer or transplantation; (2) studies focusing on acute ocular trauma, corneal injury, retinal degeneration, or optic nerve damage; (3) key comparative studies evaluating MSCs, extracellular vesicles, or alternative donor stem cell sources (including USCs).

Exclusion Criteria: Non-English publications, non-peer-reviewed conference abstracts lacking full text, and articles exclusively covering chronic non-ocular metabolic systemic conditions without translational relevance to bioenergetics or organelle transfer.

Selection of Ocular vs. Non-Ocular Studies: Primary emphasis was placed on direct ocular studies (e.g., chemical burns, optic nerve crush, and retinal ischemia–reperfusion). Due to the emerging nature of the ocular field, relevant non-ocular literature (e.g., cardiac or neural mitochondrial transplantation) was selectively included to substantiate foundational mechanisms such as macropinocytosis, TNT formation, EV packaging, and laboratory isolation protocols.

### 1.2. Bioenergetic Collapse and the Need for Bioenergetic Resuscitation in Acute Ocular Trauma

Acute ocular trauma is a leading cause of visual disability worldwide, significantly impacting both civilian and military populations [1]. These injuries, categorized as chemical, thermal, and mechanical trauma, present complex clinical challenges [2,3]. Chemical burns trigger rapid liquefactive necrosis, deep corneal penetration, and the destruction of the limbal stem cell niche [4]. Thermal injuries and mechanical injuries, ranging from penetrating lacerations to blast-induced overpressure, initiate a rapid cascade of tissue disruption and degradation [5]. In the context of modern military operations, blast-related ocular trauma accounts for up to 15% of all battlefield injuries [6]. Despite the diversity of these injuries, they share a common terminal pathway: the rapid onset of irreversible cellular apoptosis and fibrotic scarring, which can result in permanent vision loss [7].

The core of ocular trauma is a metabolic crisis [8]. Retinal and corneal cells are among the most metabolically demanding tissues in the human body. They rely heavily on oxidative phosphorylation to maintain transparency and signal transduction [9]. Following an acute injury, the ocular cellular environment is compromised by ischemia and ionic imbalances [10]. These stressors converge on the mitochondria, leading to the collapse of the mitochondrial membrane potential (ΔΨm) and a subsequent failure in ATP synthesis [10].

**Figure 1:**
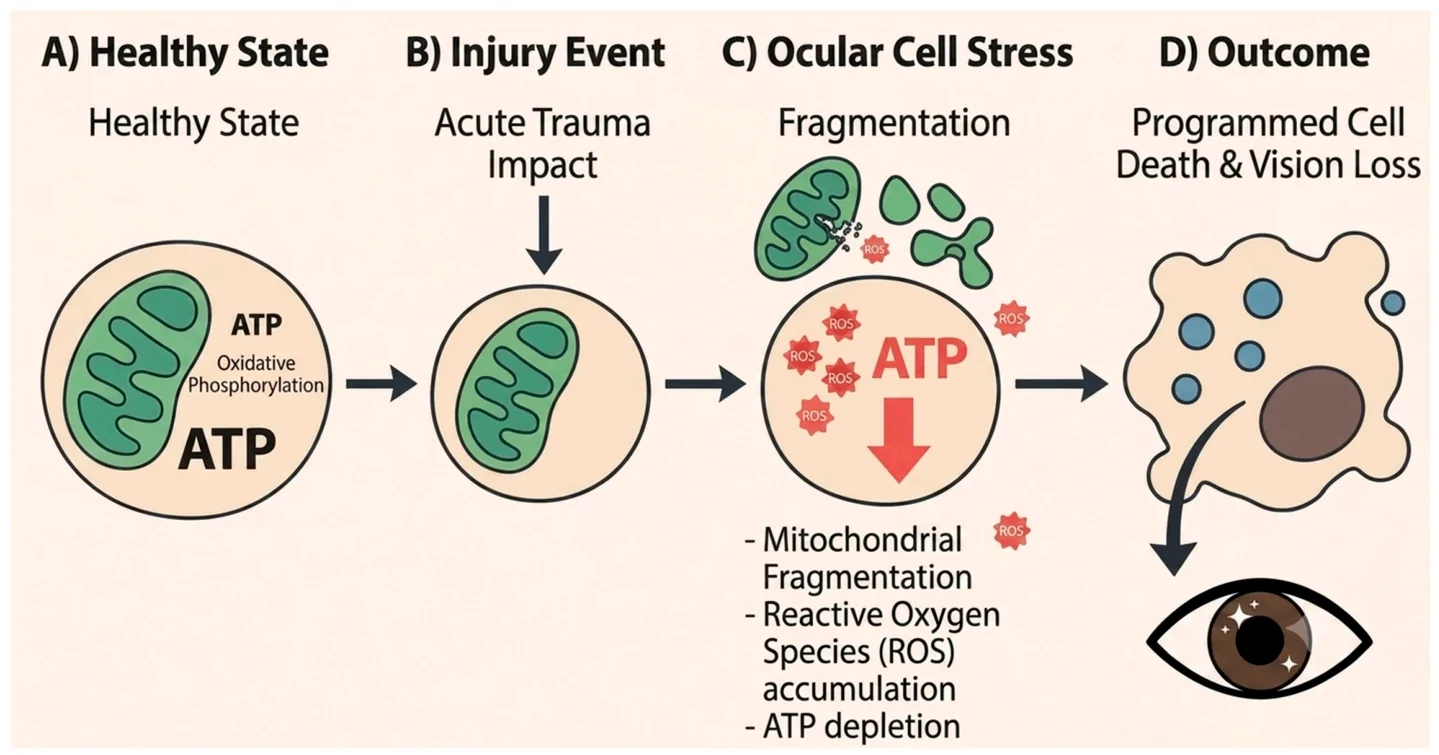
Bioenergetic collapse following acute ocular trauma. Acute injury triggers rapid intracellular ATP depletion and oxidative stress, leading to mitochondrial fragmentation and programmed cell death. (**A**). Healthy State: Mitochondria producing ATP via oxidative phosphorylation. (**B**) Injury Event: Impact of chemical/mechanical trauma. (**C**). Ocular Cell Stress: Depicted as mitochondrial fragmentation, Reactive Oxygen Species (ROS) accumulation, and ATP depletion. (**D**). Outcome: Activation of apoptotic pathways leading to vision loss.

This bioenergetic failure triggers a vicious cycle, as the depletion of ATP impairs the function of ion pumps and transporters, including those that maintain intracellular calcium (Ca^2+^) homeostasis. The resulting mitochondrial Ca^2+^ overload, together with oxidative stress, triggers opening of the mitochondrial permeability transition pore (mPTP), a mechanism discussed in detail in Section 1.3 [11]. Consequently, mitochondria release pro-apoptotic factors, such as cytochrome c, into the cytoplasm, whilst generating a burst of ROS [12]. This shifts the cellular state from survival to programmed cell death (Figure 1), suggesting that mitochondrial dysfunction is not a byproduct of trauma, but the primary driver of irreversible damage.

Current clinical protocols for acute ocular injury are primarily reactive rather than restorative. The leading standard of care focuses on suppressing inflammation through corticosteroids and preventing infection via prophylactic antibiotics [13]. While effective, these interventions do not address the underlying bioenergetic deficit the ocular cells undergo [14]. Corticosteroids, particularly, may offer symptomatic relief but can also delay epithelial healing, induce cataract, and increase intraocular pressure [15]. Furthermore, advanced surgical interventions, such as corneal transplantation or retinal surgery, are often deferred until the chronic stage of injury, by which time the regenerative capacity of the endogenous tissue is incapacitated or terminated [16]. There is currently no approved therapeutic modality that targets the immediate metabolic failure of the damaged cells. This therapeutic gap highlights the urgent need for “bioenergetic resuscitation”, a novel strategy that directly restores cellular energy production to prevent the transition from acute injury to permanent blindness.

### 1.3. Mitochondrial Calcium Overload and Permeability Transition in Acute Ocular Injury

Among the ionic disturbances that follow acute ocular injury, dysregulation of Ca^2+^ homeostasis deserves particular emphasis because it links the initial insult directly to mitochondrial failure and cell death. Under physiological conditions, mitochondria are, together with the endoplasmic reticulum (ER), one of the principal intracellular Ca^2+^ stores. Driven by the strongly negative ΔΨm, Ca^2+^ enters the mitochondrial matrix through the mitochondrial calcium uniporter (MCU) complex and is extruded mainly by Na^+^/Ca^2+^ exchange. This allows mitochondria to shape cytosolic Ca^2+^ transients and to couple Ca^2+^ signals to the activity of matrix dehydrogenases and ATP production [17].

Acute injury disturbs this balance from two directions. First, Ca^2+^ enters from the extracellular space. ATP depletion compromises the plasma membrane Ca^2+^-ATPase and the Na^+^/K^+^-ATPase, and the resulting membrane depolarization, intracellular Na^+^ accumulation, and reversal of Na^+^/Ca^2+^ exchange favour Ca^2+^ entry [10]. In the retina, ischemia additionally causes excessive release of glutamate, which activates N-methyl-D-aspartate (NMDA) receptors and voltage-gated Ca^2+^ channels and produces a large Ca^2+^ influx into inner retinal neurons [18]. Second, Ca^2+^ is released from the ER: impaired activity of the sarco/endoplasmic reticulum Ca^2+^-ATPase (SERCA) and Ca^2+^ efflux through inositol 1,4,5-trisphosphate and ryanodine receptors deplete ER stores. Because the ER is physically tethered to mitochondria at mitochondria-associated membranes, a substantial fraction of this Ca^2+^ is delivered directly to the mitochondrial surface [17]. Mitochondria initially buffer the rising cytosolic Ca^2+^, but this protective uptake becomes harmful once their capacity is exceeded [11,17].

Matrix Ca^2+^ overload, particularly in combination with oxidative stress, inorganic phosphate, and depletion of adenine nucleotides, triggers opening of the mPTP, a high-conductance, non-selective channel in the inner mitochondrial membrane [11,19]. Sustained mPTP opening collapses ΔΨm and uncouples oxidative phosphorylation, so that the ATP synthase may hydrolyse rather than produce ATP. It also causes large-amplitude swelling of the matrix, rupture of the outer mitochondrial membrane, and release of cytochrome c and other intermembrane-space proteins into the cytosol [11,19]. Released cytochrome c then activates the caspase cascade and apoptosis [12]. When ATP depletion is severe, the same mPTP-driven bioenergetic collapse leads instead to necrotic cell death, so that the extent of the permeability transition largely determines both whether and how injured cells die [11]. Moreover, because ΔΨm is the driving force for mitochondrial Ca^2+^ uptake, depolarized mitochondria lose their capacity to buffer Ca^2+^, which further amplifies cytosolic Ca^2+^ overload and perpetuates the vicious cycle described above [11,17].

This sequence is of particular importance in ophthalmology because retinal and optic nerve cells are highly Ca^2+^-sensitive. Retinal ganglion cells (RGCs) are especially vulnerable to excitotoxic Ca^2+^ influx during retinal ischemia, and in experimental models, agents that block NMDA receptors or voltage-gated Ca^2+^ channels reduce ischemic retinal damage [18]. In the optic nerve, in vivo imaging after crush injury has shown a rapid rise in intra-axonal Ca^2+^; Ca^2+^ channel blockers attenuated both this rise and the subsequent acute axonal degeneration, whereas a Ca^2+^ ionophore aggravated it [20]. In glaucomatous optic neuropathy, which shares several downstream mechanisms with traumatic optic neuropathy, Ca^2+^-dependent intracellular cascades have likewise been implicated in progressive RGC and axonal degeneration [21]. Comparable data for corneal cells after chemical injury are more limited, and the contribution of mitochondrial Ca^2+^ overload to corneal damage remains to be defined. Mitochondrial Ca^2+^ overload and mPTP opening therefore represent a point of convergence at which the ionic disturbances of ischemic and mechanical ocular injury are converted into irreversible bioenergetic failure [11,19]. Accordingly, an effective bioenergetic rescue strategy should aim not only to restore ATP production but also to re-establish mitochondrial Ca^2+^ buffering capacity. The potential of mitochondrial transplantation in this regard is discussed in Section 4.3.

## 2. Mechanisms of Mitochondrial Internalization

### 2.1. Pathways of Mitochondrial Internalization in Ocular Cells

The transfer of exogenous mitochondria into recipient cells is no longer seen as a passive occurrence but as a highly regulated biological process. In acute ocular injury, the goal is for damaged host cells to rapidly “uptake” healthy mitochondria to stop the apoptotic cascade. There are several mechanisms by which mitochondria can be internalized by recipient cells.

Macropinocytosis: Macropinocytosis is a form of endocytosis where the cell membrane folds inward to create large vesicles that engulf extracellular fluids and particles. This is the most documented pathway for “naked” mitochondrial uptake. Research suggests that when healthy mitochondria are injected, usually intravitreally, they trigger the activation of the actin cytoskeleton in damaged retinal cells, leading to their engulfment [22]. Molecules like PI3K and Rac1 are essential for this membrane remodeling.

Tunneling Nanotubes (TNTs): TNTs are long, thin, actin-based bridges that connect two or more cells over long distances. In a therapeutic setting, if MSCs are used as a delivery vehicle, these cells can extend TNTs directly into the injured ocular cells [23]. This is often more efficient than injecting isolated mitochondria because the mitochondria are pushed through the tube, protecting them from the extracellular environment.

Gap Junctions and Microvesicles: These are protein channels (e.g., connexins) that allow for the passage of smaller mitochondrial components or signals that prime the host cell for better metabolic function [24]. Often, mitochondria are packaged into microvesicles by the donor cell. These vesicles then fuse with the plasma membrane of the injured eye tissue, releasing the mitochondria directly into the cytoplasm.

The Role of the “Danger Signal”: Internalization is significantly enhanced in injured cells compared to healthy ones [25]. Acute ocular trauma releases signals (like Ca^2+^ spikes or specific chemokines) that act as a “find me” or “eat me” signal for the therapeutic mitochondria [26]. These extracellular Ca^2+^ signals, which may facilitate organelle uptake, should be distinguished from the intracellular mitochondrial Ca^2+^ overload that drives cell death (Section 1.3). This inherent targeting makes mitochondrial therapy particularly effective for localized trauma like corneal burns or optic nerve crush.

Overall, the success of mitochondrial transplantation in the ocular environment hinges on the efficiency of internalization pathways. Unlike smaller molecules, the 0.5–1.0 µm size of the mitochondrion requires active transport mechanisms [27]. Macropinocytosis remains the predominant pathway for isolated mitochondrial uptake, governed by actin polymerization and GTPase activity [28]. However, in cellular-based therapies, such as the application of stem cells to the ocular surface, the formation of TNTs provides a direct conduit for organelle transfer. Understanding these pathways is essential for optimizing delivery dosages and ensuring the metabolic rescue of the retinal and corneal bioenergetic landscapes.

### 2.2. Biological Mechanisms Governing Exogenous Mitochondrial Uptake

Multiple mechanisms have been proposed to explain the transfer of exogenous mitochondria into recipient cells, including macropinocytosis, endocytosis, membrane fusion, extracellular vesicle-mediated transport, and tunneling nanotubes (TNTs). While numerous studies have reported intracellular localization of donor mitochondrial signals following transplantation, the interpretation of these findings requires caution because the intracellular fate of transplanted mitochondria remains incompletely understood.

Fluorescent Detection Does Not Necessarily Demonstrate Functional Mitochondrial Transfer: Many studies rely on fluorescent dyes, mitochondrial-targeted fluorescent proteins, or quantum-dot labeling to track donor mitochondria [29,30]. However, detection of fluorescent signals within recipient cells does not necessarily demonstrate that intact, functional mitochondria have been successfully transplanted [31]. Fluorescent labels may persist following mitochondrial fragmentation, membrane disruption, or degradation and may potentially transfer independently of intact organelles.

Intact Organelle Uptake Versus Mitochondrial Fragments: An important unresolved question is whether recipient cells internalize complete mitochondria or merely mitochondrial fragments and associated biomolecules. Most current imaging approaches lack sufficient spatial resolution to distinguish between these possibilities.

Endocytic Uptake and Lysosomal Degradation: Macropinocytosis is frequently proposed as a major mechanism of mitochondrial internalization. However, uptake via endocytic pathways raises the possibility that transplanted mitochondria may subsequently traffic to lysosomes and undergo degradation rather than contribute to host–cell metabolism.

Mitochondrial Fusion, Fission, and Network Incorporation: Functional incorporation into host cells likely requires participation in normal fusion-fission dynamics regulated by proteins such as MFN1, MFN2, OPA1, and DRP1. Evidence demonstrating direct fusion between donor and host mitochondria remains limited in ocular systems.

Donor mtDNA Persistence, Heteroplasmy, and Mitophagy: Few ocular studies have examined mtDNA durability, heteroplasmy, or mitophagic clearance of transplanted mitochondria after uptake.

Defining Functional Integration: For the purposes of this review, functional integration is defined as meeting several criteria: (1) intracellular uptake of intact donor mitochondria; (2) evasion of lysosomal degradation; (3) participation in host mitochondrial network dynamics; (4) restoration or enhancement of oxidative phosphorylation, ATP production, or mitochondrial membrane potential; (5) persistence of donor mitochondrial function beyond transient intracellular detection; and (6) evidence of therapeutic benefit attributable to donor mitochondrial activity rather than indirect signaling effects.

## 3. Sources and Delivery Methods

The efficacy of mitochondrial transplantation in the eye is largely dependent on the quality of the donor source and the precision of the delivery route. While various cell types have been explored, mesenchymal stem cells (MSCs) are emerging as a superior candidate for organelle donation.

### 3.1. Mesenchymal Stem Cells as Optimal Mitochondrial Donors

MSCs as a Potent Mitochondrial Donor: MSCs are a promising source for mitochondrial transplantation due to their accessibility, biological resilience, and capacity for intercellular organelle transfer. Traditionally, they are derived from bone marrow, adipose tissue, and perinatal tissues [32]. However, MSC-like populations can be isolated from non-invasive sources such as urine, increasing accessibility [33]. This circumvents the need for procedures like bone marrow aspiration, enabling repeated sampling and the potential establishment of patient-specific “mitochondrial banks” [34]. It also reduces immunogenic risks and allows for personalization of therapeutic strategies [35]. For acute ocular injury rapid intervention is critical [36]. The accessibility of MSCs facilitates the timely delivery of therapeutic mitochondria.

MSCs’ robust proliferative potential and sustained telomerase activity compared to differentiated somatic cells [37] permits efficient in vitro expansion of MSC without premature cellular aging, yielding a higher count of functional mitochondria. These MSC-isolated mitochondria tend to exhibit preserved membrane potential and efficient oxidative phosphorylation [38], and when transplanted they have been shown to reduce ROS accumulation, restore oxidative phosphorylation, and improve cellular survival in injured tissues [39]. Beyond transplantation of isolated mitochondria, MSCs are capable of active mitochondrial transfer through TNTs, EVs, or direct cell–cell contact [40]. This capability highlights their biological compatibility as mitochondrial donors and suggests that exogenously isolated mitochondria retain functional properties optimized for cellular uptake and integration.

### 3.2. Comparative Analysis: Isolated Organelle Injection vs. Whole Cell-Based Transfer

There are two primary strategies for using MSC in mitochondrial transplantation: injection of isolated mitochondria and direct MSC transplantation. Both approaches aim at restoring cellular bioenergetics in damaged ocular tissue, yet they differ in mechanisms, timing, and complexity.

For injection of isolated mitochondria, mitochondria are extracted from MSCs using several purification and centrifugation protocols [41]. The isolated mitochondria are then suspended in a respiration-promoting buffer and delivered directly into the ocular tissue through an intravitreal injection. This delivery allows for rapid mitochondrial uptake that immediately enhances the bioenergetics of the recipient cells [42]. However, this strategy is strongly dependent on mitochondrial viability post isolation. Once mitochondria are removed from their native environment, they become particularly vulnerable to oxidative stress and efficient cellular uptake is not guaranteed. Hence, injected mitochondria can provide rapid intervention yet may be limited due to its transient effects.

Another strategy is the direct transplantation of MSC into the ocular tissue. In this situation, MSC work as a responsive mitochondrial donor, reacting to local tissue injury and actively transfer mitochondria to damaged cells through TNTs, gap junctions, and EVs [38]. This approach allows for sustained mitochondrial transfer and additional cytoprotective benefits, including the secretion of anti-inflammatory cytokines. MSCs have an intrinsic mitochondrial quality control system that ensures the transfer of functional organelles. However, the survival and behavior of transplanted MSCs within the ocular environment is variable and potentially influenced by immune response.

Overall, both strategies provide promising approaches to mitochondrial transplantation as a therapeutic strategy in cases of ocular injury. The choice between them is predominantly dependent on clinical context and therapeutic timeline, as MSC-mediated delivery offers a more sustained long-term approach whilst injection of isolated mitochondria provides a more rapid intervention.

**Figure 2:**
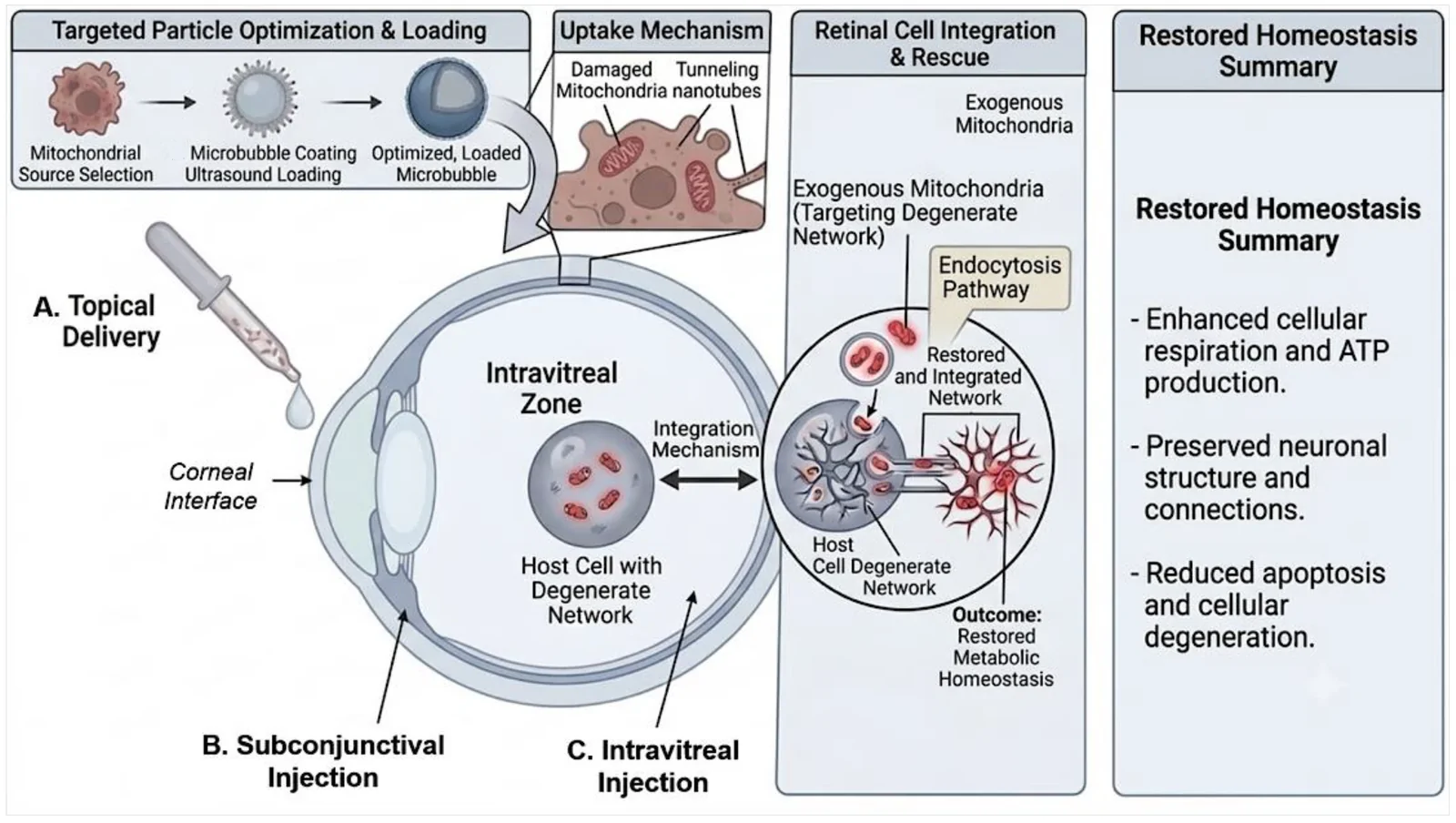
Schematic Overview of Mitochondrial Delivery Strategies, Uptake Mechanisms, and Therapeutic Integration for Retinal Rescue. (Top Left) Target particle optimization and loading workflow, detailing mitochondrial source selection and subsequent microbubble coating via ultrasound loading to yield optimized, loaded microbubbles. (Bottom Left) Clinical routes for therapeutic delivery to the posterior segment, highlighting (A) Topical Delivery at the corneal interface, (B) Subconjunctival Injection, and (C) Intravitreal Injection into the intravitreal zone to reach host cells exhibiting degenerate networks. Notably, cellular stress and progressive mitochondrial dysfunction induce targeted degradation of tight junctions, resulting in severe damage to the blood-retinal barrier (BRB). This compromised barrier state alters local pharmacokinetics and tissue penetrability during delivery, providing an important pathological context for route selection [43]. (Top Center) Cellular uptake mechanisms, depicting the internalization of exogenous mitochondria into recipient cells via endocytosis pathways and physical transfer through tunneling nanotubes connecting healthy and damaged cells. (Right Center) Retinal cell integration and structural rescue, wherein internalized exogenous mitochondria migrate toward the host cell’s degenerate network. This integration rescues metabolic signaling, forming a restored, highly integrated metabolic network that preserves overall tissue structure. (Far Right) Downstream functional outcomes of successful mitochondrial transplantation and network repair, culminating in restored metabolic homeostasis characterized by: (i) enhanced cellular respiration and optimized ATP production, (ii) preservation of neuronal structure and intercellular connections, (iii) systemic reduction in apoptosis and cellular degeneration, and (iv) functional stabilization and rescue of the damaged blood-retinal barrier.

*Ocular Delivery Routes for Acute Injury* (Table 1 *and *Figure 2): The eye’s unique anatomy allows for targeted delivery depending on the site of the acute injury. Route selection for the posterior segment must also take into account the integrity of the blood–retinal barrier (BRB). As illustrated in Figure 2, mitochondrial dysfunction can compromise this barrier: in human retinal pigment epithelial (ARPE-19) cells exposed to high glucose, mitochondrial dysfunction was accompanied by loss of outer BRB integrity, and pharmacological antagonism of the α1D adrenergic receptor protected against both [43]. A compromised BRB may, in turn, alter the local pharmacokinetics and tissue penetration of intravitreally or subretinally delivered mitochondria.

### 3.3. Non-Invasive Procurement: Urine-Derived Stem Cells as an Emerging Source

Selecting an optimal donor source is critical for the success of mitochondrial transplantation. Urine-derived stem cells (USC) represent a paradigm shift in this regard due to their ready availability, scalability, ethics, and non-invasive procurement of robust mitochondria. When compared to other mesenchymal sources, USCs demonstrate superior expansion rates and a greater metabolic profile [35]. In the context of acute ocular trauma, these cells can be utilized either as a source for isolated organelle extraction or as live delivery vehicles. By leveraging routes such as intravitreal or subconjunctival injection, USC-derived mitochondria can be delivered directly to the site of metabolic collapse, offering a rapid intervention to prevent irreversible vision loss.

### 3.4. Ocular Delivery Routes and Critical Evaluation of Translational Evidence

Topical Administration: Topical delivery currently has the strongest evidence for anterior segment applications (e.g., corneal epithelial repair). However, significant biological questions remain regarding mitochondrial stability within tear fluid, interactions with mucins, residence time, dose reproducibility, and penetration beyond the epithelial barrier.

Subconjunctival Administration: Subconjunctival injection is frequently proposed, but direct evidence demonstrating successful delivery of isolated mitochondria into the corneal stroma, limbal stem-cell niche, or intraocular tissues is currently lacking (conceptual/extrapolated).

Intravitreal & Subretinal Administration: Intravitreal delivery has been demonstrated in preclinical retinal and RGC models, yet stability in vitreous humor, cell-type specificity, clearance kinetics, and inflammatory risks require further characterization.

## 4. Applications for Specific Ocular Pathologies and Evidence Stratification

Acute ocular injuries trigger a cascade of bioenergetic failure characterized by acute ATP depletion, mitochondrial Ca^2+^ overload with mPTP opening, membrane potential collapse, and oxidative stress. However, current clinical management of acute trauma focuses on stabilization rather than cellular metabolic rescue. Standard clinical protocols entail immediate copious irrigation and pH normalization for chemical insults, combined with cycloplegia, matrix metalloproteinase inhibition (e.g., topical tetracyclines or citrate), anti-inflammatory modulation, and prophylactic antimicrobial coverage, followed by staged reconstructive surgical procedures when necessary. In addition to corticosteroids and conventional NSAIDs, emerging therapies seek to simultaneously target inflammation and oxidative stress. Pittalà et al. [44] developed hybrid compounds combining caffeic acid phenethyl ester (CAPE), a naturally occurring antioxidant and anti-inflammatory molecule, with traditional NSAIDs. In an experimental ocular inflammation model, CAPE-NSAID hybrids (particularly a CAPE–aspirin derivative) significantly reduced clinical inflammatory scores and decreased levels of PGE_2_ and TNF-α in aqueous humor. Although these compounds do not involve mitochondrial transplantation, they illustrate how modulation of the inflammatory and oxidative microenvironment may complement future regenerative organelle therapies.

To rigorously evaluate the therapeutic potential of mitochondrial transfer, evidence must be stratified by direct ocular testing, non-ocular model extrapolation, mechanistic plausibility, and speculative clinical translation.

### 4.1. Direct Evidence from Ocular Models

Corneal Epithelium: Direct application of isolated mitochondria or stem cell co-culture in corneal injury models demonstrates uptake by damaged corneal epithelial cells. Exogenous organelle delivery accelerates epithelial wound closure, restores cellular energy, and attenuates myofibroblast-driven fibrotic scarring [41].

Retinal Ganglion Cells (RGCs) & Optic Nerve: RGCs are acutely vulnerable to traumatic optic neuropathy (TON) and optic nerve crush injuries [45], owing to their exceptionally high metabolic demands [8,46]. Preclinical intravitreal administration demonstrates that exogenous mitochondria are internalized by RGCs, preserving cell viability, maintaining axonal integrity, and stabilizing oxidative phosphorylation [47].

Retinal Ischemia–Reperfusion (I/R) Injury: Acute intraocular pressure (IOP) spikes or vascular occlusion models induce severe transient ischemia followed by reperfusion-induced ROS bursts [18,48]. Direct mitochondrial transplantation during or immediately following the ischemic window functions as a metabolic and antioxidant buffer, significantly reducing retinal tissue damage/infarction and preserving electrophysiological function on electroretinography (ERG).

### 4.2. Evidence Derived from Non-Ocular Tissues

Given that mitochondrial transplantation in ophthalmology is an emerging field, key foundational mechanisms and organelle transfer proofs-of-concept are adapted from established non-ocular injury models:

Cardiac Models: Ischemia–reperfusion and myocardial infarction models demonstrate that direct injection of autologous mitochondria restores ATP, reduces infarct size, and recovers mechanical contraction [42,49].

Neurologic & Spinal Cord Models: Focal cerebral ischemia and spinal cord crush studies confirm that exogenous mitochondrial uptake reduces neuroinflammation, preserves neuronal integrity, and restores locomotor function [50,51].

Pulmonary Models: Acute lung injury models demonstrate that mesenchymal stem cell (MSC)-mediated transfer via microvesicles or tunneling nanotubes (TNTs) mitigates alveolar–capillary barrier disruption [52].

### 4.3. Mechanistic Plausibility

Across both ocular and systemic systems, the primary bioenergetic rationale for mitochondrial transfer rests on four core pillars:

ATP Restoration: Bypassing damaged endogenous electron transport chain complexes to rapidly restore oxidative phosphorylation.

ROS Reduction: Suppressing excessive reactive oxygen species production and clearing oxidative stress markers.

Membrane Potential Rescue: Re-establishing the mitochondrial membrane potential (ΔΨ_m) to inhibit Cytochrome c release and block downstream caspase-dependent apoptotic cascades.

Ca^2+^ Buffering and mPTP Stabilization: Because mitochondrial Ca^2+^ uptake is driven by ΔΨm, a pool of transplanted, well-polarized mitochondria could, in principle, increase the capacity of injured cells to sequester excess cytosolic Ca^2+^ and limit the matrix Ca^2+^ overload that triggers mPTP opening in endogenous mitochondria [17,19]. Experimental support for this concept currently comes from muscle pathology. In dystrophin-deficient mdx mice, which exhibit chronic Ca^2+^ dysregulation, repeated intramuscular injection of mitochondria isolated from healthy muscle reduced mitochondrial Ca^2+^ overload and Ca^2+^ deposits in skeletal muscle, and the authors proposed that the transplanted organelles take up excess Ca^2+^; notably, the treatment did not restore the Ca^2+^ retention capacity of the host mitochondria [53]. Whether transplanted mitochondria can normalize Ca^2+^ homeostasis in corneal, retinal, or optic nerve cells has not yet been tested, and the possibility that donor mitochondria themselves become Ca^2+^-overloaded and undergo permeability transition in the post-injury environment must also be considered. Future ocular studies should therefore include direct measurements of cytosolic and mitochondrial Ca^2+^, mitochondrial Ca^2+^ retention capacity, and mPTP opening alongside conventional bioenergetic endpoints.

### 4.4. Proposed or Speculative Clinical Applications

While acute trauma represents the primary experimental target, mitochondrial transplantation is conceptually proposed for chronic or complex degenerative conditions:

Ocular Surface Reconstruction: Restoring chronic limbal stem cell deficiency following severe chemical or thermal burns [54,55].

Glaucomatous Neuroprotection: Supplementing chronic bioenergetic deficits in RGCs subject to persistent IOP stress [21,46].

Degenerative Retinal Pathologies: Proposed applications in Age-Related Macular Degeneration (AMD) and Diabetic Retinopathy to mitigate chronic RPE and vascular endothelial degeneration.

### 4.5. Ocular Compartments with Limited or No Direct Evidence

To maintain scientific rigor, it is critical to highlight that several anatomical compartments currently lack direct in vivo evidence for isolated mitochondrial transplantation (Table 2 and Table 3):

Limbal Stroma & Deep Corneal Stroma: Lacks established delivery and engraftment data.

Aqueous Outflow Pathways (Trabecular Meshwork): No direct organelle rescue demonstrated.

Crystalline Lens: No evidence of functional organelle transfer.

Photoreceptors: Direct in vivo functional integration in outer retinal layers following isolated organelle delivery remains limited and unproven.

Secondary Traumatic Complications: Distinct mechanical shockwave injuries (such as commotio retinae) must be uncoupled from vascular I/R models. Furthermore, ocular siderosis represents a delayed chemical toxicity resulting from retained iron foreign bodies rather than an immediate acute trauma target [56].

**Table 2:** Direct Ocular Evidence for Mitochondrial Transplantation.

Key Limitations	Follow-Up Duration	Primary Outcome	Tracking Method	Delivery Route	Donor Source	Study Type (In Vitro/In Vivo)	Condition/Model
Limited stromal penetration; transient functional durability	Short-term (Days)	Accelerated epithelial wound closure; reduced oxidative stress	Fluorescent Dyes (MitoTracker)	Topical/Contact Lens	MSCs/Primary Cells	In Vitro & In Vivo (Rodent)	Corneal Epithelial Injury/Burn
Non-specific cellular uptake; potential vitreous inflammatory response	Short to Medium (1–4 Weeks)	Increased RGC survival; attenuated axonal degeneration	Transgenic/Mito-Fluorescent Label	Intravitreal Injection	MSCs/Exogenous Organelles	In Vivo (Rodent)	Optic Nerve Crush/TON
Unclear long-term organelle fate and fusion dynamics	Short-term (Days to 2 Weeks)	Reduced retinal tissue damage/infarction; ERG response preservation	Nanoparticle/Dye Labeling	Intravitreal Injection	Isolated Mitochondria/MSCs	In Vivo (Rodent)	Retinal Ischemia–Reperfusion

**Table 3:** Non-Ocular Evidence Relevant to Ocular Translation.

Relevance to Ocular Translation	Key Functional Findings	Uptake Mechanism	Primary Donor Source	System/Model Type
Establishes feasibility of direct organelle injection during acute ischemic events.	ATP recovery; reduced infarct size; functional contraction rescue	Macropino cytosis	Autologous Tissue/MSCs	Cardiac (Myocardial Ischemia)
Demonstrates protection of metabolically high-demand CNS neurons analogous to RGCs.	Neuroprotection; suppressed neuroinflammation; behavioral recovery	TNTs & Micro vesicles	Neural Stem Cells/MSCs	Neurologic (Cerebral Ischemia / CNS)
Highlights EV- and TNT-mediated organelle transfer mechanisms.	Restored alveolar bioenergetics; reduced vascular permeability	Micro vesicles/Direct TNTs	MSCs	Pulmonary (Acute Lung Injury)
Demonstrates axonal preservation following acute mechanical crush injury.	Motor neuron preservation; locomotor functional improvement	Endocytosis	Isolated Mitochondria	Spinal Cord Trauma

## 5. Lab Processing of MSC for Mitochondrial Therapy

The workflow for preparing MSC-derived mitochondria involves three critical phases: Isolation and Expansion, Mitochondrial Extraction, and Quality Control.

MSC Isolation and Expansion (The Source): MSCs can be isolated from several tissue sources, including bone marrow, adipose tissue, umbilical cord tissue, and urine-derived stem cells [57]. Following isolation, MSCs are cultured under controlled conditions and expanded to generate sufficient cellular biomass for mitochondrial harvesting. Expansion is typically limited to early passages (approximately 3–5) to minimize cellular senescence and preserve mitochondrial function [58]. Maintaining low-passage MSC populations is important, as prolonged culture can impair oxidative phosphorylation efficiency and increase mitochondrial dysfunction.

Mitochondrial Extraction (The Product): Once MSC cultures reach approximately 80–90% confluence, mitochondria are isolated while preserving membrane integrity and respiratory capacity [59]. Differential centrifugation remains the most widely utilized method for mitochondrial extraction due to its relative simplicity and ability to generate highly enriched mitochondrial fractions.

Initially, MSCs are homogenized in an isotonic, sucrose-based isolation buffer to disrupt the plasma membrane while maintaining mitochondrial structural integrity [60]. The homogenate subsequently undergoes sequential centrifugation steps. Low-speed centrifugation removes nuclei and cellular debris, while higher-speed centrifugation pellets mitochondria. The resulting mitochondrial fraction is purified and resuspended in cold respiration-supporting buffer (e.g., MiR05) to preserve membrane potential and metabolic activity until transplantation [61]. Preservation of the outer and inner mitochondrial membranes throughout extraction is critical, as disruption of membrane integrity impairs oxidative phosphorylation and ATP generation capacity.

Quality Control (The Validation): Prior to transplantation, isolated mitochondria must undergo rigorous quality control to confirm their viability, purity, and bioenergetic competence [62]. Functional validation commonly includes assessment of the oxygen consumption rate (OCR) using Seahorse XF analysis, which evaluates mitochondrial respiratory activity and oxidative phosphorylation capacity [63]. In parallel, mitochondrial membrane potential is measured using fluorescent probes such as JC-1 staining, providing insight into membrane polarization and overall organelles viability [64]. Purity analyses are also performed to verify enrichment of mitochondrial fractions while excluding contamination from other cellular organelles. This is commonly achieved through Western blotting for mitochondrial markers such as TOM 20 or COX IV, alongside the absence of non-mitochondrial proteins such as Calnexin, an endoplasmic reticulum marker [65]. Collectively, these quality control measures ensure that transplanted mitochondria retain sufficient structural integrity and metabolic function to appropriately support cellular recovery following acute ocular injury.

Overall, laboratory processing of MSC-derived mitochondria represents a scalable pipeline for mitochondrial therapeutics. Continued refinement of these protocols will be essential for improving reproducibility and advancing clinical translation.

## 6. Advantages of Mitochondrial Transplantation

Stem cell therapy, exosome or EV therapy, and mitochondrial transplantation address distinct pathological processes following acute ocular injury. When comparing these three modalities, transplantation of MSC-derived mitochondria proves advantageous due to a safety profile similar to that of exosome therapy, as isolated mitochondria lack nuclei and hence can’t form tumors [66], and the ability to provide immediate bioenergetic intervention critical due to the time-sensitive nature of acute ocular injury-triggered metabolic cascades that lead to permanent vision loss (Table 4 and Table 5).

**Table 4:** Comparative Overview of Regenerative Modalities.

Mitochondrial Transplantation	Cell-Free Therapy [67] (Exosome or EV)	Stem Cell Therapy (MSC)	Feature
Intact metabolic organelles	Lipid vesicles with RNA/proteins	Living, multipotent cells	Active Component
Bioenergetic rescue & ATP restoration	Intercellular signaling & modulation	Tissue replacement & paracrine signaling	Primary Goal
Rapid (immediate ATP boost)	Moderate (signaling cascade)	Slow (requires integration/ differentiation)	Onset of Action
Low (if autologous /MSC-derived)	Low (acellular)	Moderate (risk of rejection)	Immuno- genicity
High (no nuclear DNA/proliferation)	High (no DNA/proliferation)	Risk of tumorigenesis/ mal-differentiation	Safety
Survival of high-metabolic cells (RGC)	Anti-inflammatory “quieting”	Long-term tissue scaffolding	Ocular Advantage

Abbreviations: ATP: Adenosine Triphosphate (the primary energy currency of the cell), EV: Extracellular Vesicle, MSC: Mesenchymal Stem Cell, RGC: Retinal Ganglion Cell, RNA: Ribonucleic Acid.

Stem Cell Therapy: Stem cell therapies are a viable strategy due to their capacity for long-term survival within damaged tissues and continued secretion of growth factors [68]. Unlike acellular therapies, MSCs possess multipotent capabilities. Hence, they can contribute to regeneration of injured corneal and retinal structures in cases of severe trauma. However, these advantages are accompanied by notable limitations. Whole-cell transplantation carries risks associated with uncontrolled proliferation, aberrant differentiation, and tumorigenesis [69]. Furthermore, logistical challenges related to cell preservation, storage, and transplantation further complicate clinical implementation [70].

Exosome Therapy: Exosome therapies represent a cell-free alternative that functions primarily through intercellular signaling and immunomodulation [71,72]. As exosomes lack proliferative capacity, they offer improved safety with minimal risk of tumor formation. Their nanoscale size facilitates penetration through ocular barriers, enabling efficient delivery of proteins, lipids, and regulatory RNAs that mitigate inflammation and promote tissue repair [73]. They are also particularly efficient at mitigating cytokine storms [74]. Nevertheless, exosomes are unable to directly replace damaged tissue, instead relying on surviving host cells to mediate repair processes. Additionally, rapid clearance by lymphatic system may limit therapeutic duration, causing repeated administration to sustain beneficial effects [75].

Mitochondrial Transplantation: Mitochondrial transplantation distinguishes itself from other regenerative modalities by directly addressing cellular bioenergetic dysfunction [76]. In acute ocular injury, cells undergo rapid degeneration. Transplanted mitochondria may restore metabolic capacity, stabilize membrane potential, and reduce apoptotic signaling, hence preserving RGC before irreversible injury occurs. Similar to exosome therapies, isolated mitochondria provide a cell-free intervention. Moreover, mitochondrial therapies exhibit an exceptionally rapid onset of action, potentially restoring cellular metabolism shortly after administration [77]. Despite these advantages, mitochondrial transplantation faces important challenges. Isolated mitochondria are very fragile outside the intracellular environment [78]. Standardization of dosing, delivery efficiency, and long-term functional outcomes also remains unclear, highlighting the need for continued investigation before widespread clinical adoption.

**Table 5:** Distinguishing characteristics of regenerative modalities in ophthalmology. While traditional cell therapies rely on long-term integration, mitochondrial transplantation offers a unique bioenergetic intervention suitable for the narrow therapeutic window following acute injury. While stem cells are great for long-term reconstruction, they are often too slow for the metabolic emergency of acute trauma. Exosomes are excellent at halting inflammation, but mitochondria are the only modality that restores the bioenergetics of the dying cells.

Mitochondrial Transplantation	Cell-Free Therapy (Exosome- or EV-Based)	Stem Cell Therapy (MSC-Based)	Feature
Functional organelles (0.5–1.0 mum)	Nano-vesicles (30–150 nm)	Whole multipotent cells	Active Biological Unit
Direct Bioenergetic Rescue	Transfer of miRNA and proteins	Paracrine signaling & niche modulation	Primary Mechanism
Acute ATP depletion/ Metabolic crisis	Inflammatory “cytokine storm”	Chronic tissue remodeling	Target in Ocular Trauma
Rapid (minutes to hours)	Intermediate (hours to days)	Delayed (days to weeks)	Speed of Benefit
Moderate	Low	High	In Vivo
(requires intracellular uptake)	(Rapidly cleared)	(can engraft)	Stability
Minimal (MHC-independent)	Minimal (Low immunogenicity)	Variable (MHC expression)	Immunogenicity
None (Acellular)	None (Acellular)	Potential (teratoma/hyperplasia)	Risk of Malignancy
Very High (Requires “Cold Chain” +4 °C)	High (Lyophilization possible)	Complex (Cryo- preservation/Thawing)	Storage & Logistics
Traumatic Optic Neuropathy & Ischemia	Chemical burns & Uveitis	Limbal Stem Cell Deficiency	Ideal Clinical Use Case

Abbreviations: MSC: mesenchymal-derived Stem Cell, EV: extracellular Vesicle, and ATP: adenosine Triphosphate.

## 7. Strategic Selection of Stem Cell Sources for Mitochondrial Transplantation: The Rising Potential of USCs

The success of mitochondrial transplantation is influenced mainly by its delivery route, processing methods, and the biological characteristics of the donor cell source. To date, adipose-derived mesenchymal stem cells (AD-MSCs) and bone marrow-derived mesenchymal stem cells (BM-MSCs) have served as the main reservoirs for mitochondrial isolation due to their established clinical use and high cellular yield [79]. Adipose tissue offers a substantial initial biomass, allowing for efficient harvesting of mitochondria from large numbers of MSCs post liposuction. Bone marrow-derived MSCs remain a well-characterized source but require invasive aspiration and tend to exhibit lower stem cell frequency compared to adipose tissue.

Recently, placental and umbilical cord-derived MSCs have gained attention as alternative donor sources due to their developmental immaturity and low accumulation of mtDNA damage [80]. Mitochondria derived from these tissues may exhibit enhanced respiratory capacity and reduced oxidative burden relative to adult-derived sources. This suggests potential advantages for regenerative applications requiring maximal bioenergetic performance.

In contrast to other donors, urine-derived stem cells (USCs) are a particularly accessible and minimally invasive alternative [81]. USCs can be obtained repeatedly through routine urine collection without surgical intervention. This accessibility raises the possibility of creating patient-specific mitochondrial repositories and therapies. Furthermore, repeated collection enables longitudinal sampling, potentially allowing optimization of donor mitochondrial quality prior to transplantation.

While traditional sources have established benchmarks for mitochondrial yield, USCs are emerging as a highly promising, non-invasive and autologous alternative. Though comparative studies are currently limited, early research suggests that USCs offer unique metabolic advantages and bypass allogeneic rejection that could redefine personalized regenerative medicine.

### Key Technical Considerations (Table 6)

1. Scalability and Proliferative Power: A primary consideration in donor source selection is the balance between initial mitochondrial yield and long-term scalability. Adipose-derived MSC provide exceptionally high starting biomass, enabling rapid isolation of large mitochondrial quantities with minimal expansion requirements [82]. While USCs populations initially have fewer stem cells but possess improved proliferative potential in proper culture. Following expansion, USC cultures may generate therapeutically relevant mitochondrial quantities while preserving the advantage of recipient-specificity and repeated, non-invasive collection.

**Table 6:** Comparison of Sources for Mitochondrial Isolation.

Placenta/Cord	Bone Marrow (BM)	Adipose (Fat)	USC (Urine)	Feature
Easy (Post-birth)	Difficult (Painful Aspiration)	Moderate (Liposuction)	Easiest (Non-invasive)	Ease of Collection
High	Low	Highest	Abundant, ~1–5 stem cell clones/100 mL)	MSC Frequency
Variable	High	High	High	Mito Transfer Efficiency
Superior (Young)	Stable (Standard)	Good (Abundant)	High (Metabolic focus)	Mitochondrial Quality
Moderate	Moderate	Easiest (Bulk tissue)	High (via cell expansion)	Scalability
2–4 Weeks	2–4 Weeks	2–3 weeks	2–3 Weeks	Cultured cells for Time-to-Dose

Abbreviations: BM: Bone Marrow, MSC: Mesenchymal Stem Cell, Mito: Mitochondrial, USC: Urine-Derived Stem Cell.

2. Intrinsic Transfer Capacity: Effective mitochondrial transplantation depends not only on mitochondrial abundance but also on the ability of donor cells to support organelle transfer and uptake. Proteins such as Miro1, involved in mitochondrial trafficking, and Connexin-43 (Cx43), involved in intercellular transfer mechanisms, have been associated with enhanced mitochondrial transfer efficiency in MSC populations [83]. Although AD-MSCs and BM-MSCs have established transfer capabilities, little is known about expression profiles for USC populations. Given their distinctive metabolic phenotype and secretory characteristics, USCs may exhibit unique transfer kinetics and altered interactions with recipient tissues. Future investigations comparing Miro1 and Cx43 expression among donor sources could clarify whether USC-derived autologous mitochondria possess enhanced therapeutic target potential without untoward immunogenicity.

3. Mitochondrial Fitness and Donor Optimization: Placental and umbilical cord-derived tissues provide “young” mitochondria characterized by lower cumulative oxidative damage and reduced mtDNA mutation burden. However, these sources are limited by availability and reduced suitability for autologous transplantation. USCs may offer a distinct advantage through compatibility with personalized autologous therapies, minimizing immune incompatibility while enabling repeated donor sampling. As USC populations require expansion prior to therapeutic application, this culture period presents the opportunity for mitochondrial preconditioning strategies designed to optimize respiratory function, stress resistance, or metabolic performance before transplantation.

4. The “Time-to-Dose” Evolution: Traditional donor sources may provide shorter time-to-dose intervals due to immediate availability of large tissue volumes. In contrast, USC-derived therapies generally require an expansion period before sufficient mitochondrial quantities are obtained. Although this delay may reduce utility in emergency interventions, it may prove advantageous for planned or personalized treatments. Consequently, the optimal donor source may depend less on absolute superiority and more on clinical context. Acute emergencies may favor immediately available adipose-derived mitochondria, whereas chronic or personalized applications may benefit from the scalability and accessibility of USCs.

## 8. Challenges and Future Directions

While the therapeutic potential of MSC-derived mitochondrial transplantation is immense, several hurdles must be addressed to transition this therapy from the laboratory to the clinic. Mitochondria are highly sensitive organelles that when isolated begin to lose functional viability rapidly if not kept on ice in specialized buffers. Preservation of mitochondrial integrity during storage and transport is a significant translational barrier. Developing cryopreservation techniques is necessary to tackle this issue. Lyophilization, nanocarrier encapsulation, and biomaterial-assisted stabilization are currently being investigated as methods to prolong mitochondrial functionality and improve delivery efficiency.

Another major challenge is the lack of standardized dosing metrics for mitochondrial therapies. In conventional pharmacology, dosing is typically quantified in milligrams, whereas cell therapies rely on total cell counts. By contrast, mitochondrial transplantation lacks a universally accepted unit capable of correlating quantity with therapeutic efficacy. Establishing a reproducible potency assay will be critical for ensuring consistent clinical outcomes across patient populations and types of injury.

Additionally, confirming successful mitochondrial uptake and long-term persistence within target ocular tissues is difficult. Distinguishing transplanted mitochondria from endogenous host mitochondria in vivo is challenging, particularly over extended periods following transplantation. Current preclinical studies rely on fluorescent labeling systems and quantum dot tracking to monitor mitochondrial trafficking, integration, and long-term metabolic contribution within recipient cells. Improved imaging modalities and real-time tracking technologies may become essential tools for validating therapeutic efficacy.

Although MSCs have low-immunogenicity, introduction of “naked” exogenous mitochondria may trigger a localized inflammatory response. MtDNA and cardiolipin can act as damage-associated molecular patterns (DAMPs) that activate innate immune signaling pathways [84]. Hence, decreasing inflammatory recognition of transplanted mitochondria is an important objective. One emerging strategy involves utilizing MSC-derived EVs as a delivery system capable of shielding mitochondria from immune surveillance while simultaneously improving cellular uptake and tissue targeting.

### Regulatory and Ethical Pathways

**Figure 3:**
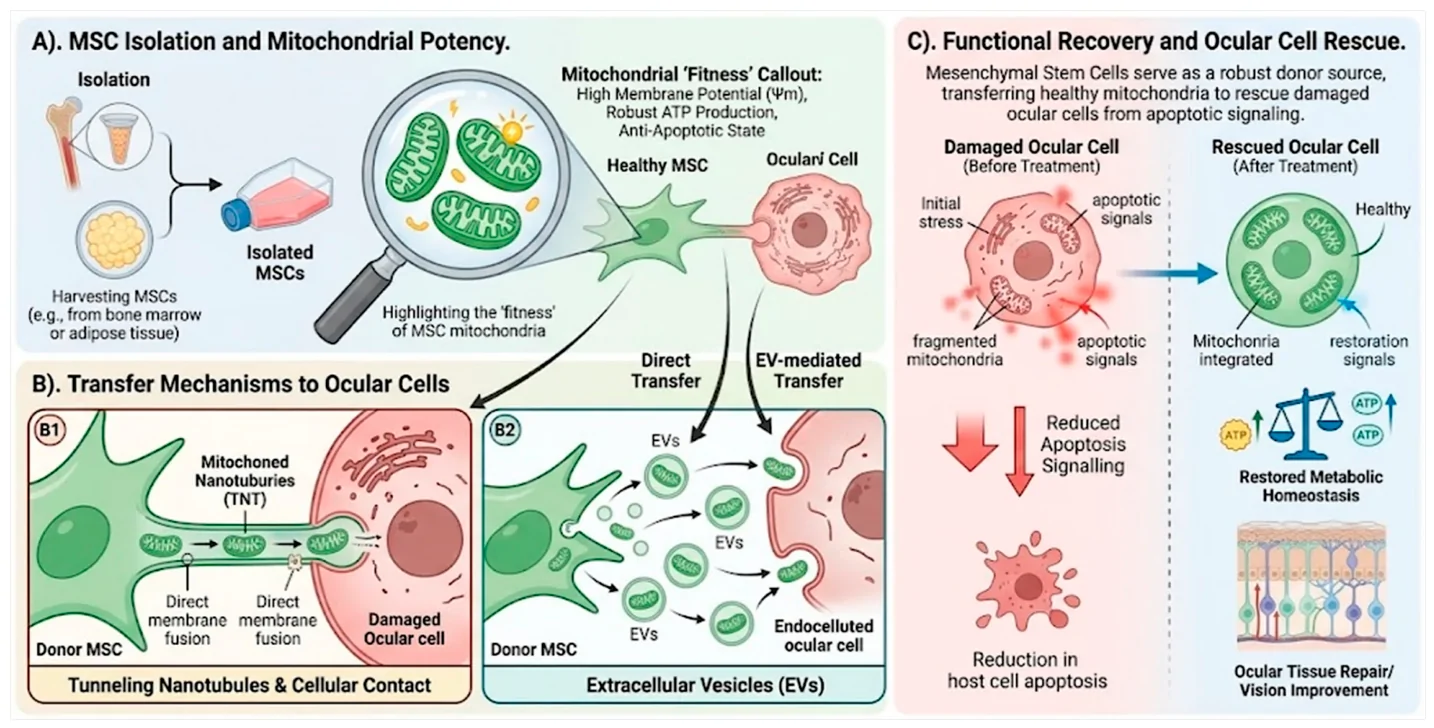
Mesenchymal Stem Cells (MSCs) as a potent source for mitochondrial transplantation. Mesenchymal Stem Cells serve as a robust donor source, transferring healthy mitochondria to rescue damaged ocular cells from apoptotic signaling. (**A**). MSC Isolation: harvesting MSCs (e.g., from bone marrow or adipose tissue) and Mitochondrial Potency: highlighting the “fitness” of MSC mitochondria (high membrane potential). (**B**). Transfer Mechanisms: showing MSCs donating mitochondria directly to ocular cells via extracellular vesicles (EVs) or direct cellular contact, nanotubules and extracellular vesicles (**C**). Functional Recovery: reduction in host cell apoptosis and restoration of metabolic homeostasis.

Mitochondrial transplantation faces a complex regulatory landscape. Determining whether isolated mitochondria should be classified as a biological, cellular therapy, or tissue-derived product under FDA and EMA frameworks remains unclear. Large-scale clinical translation requires establishing standards for manufacturing practices, donor screening protocols, sterility validation, and long-term safety monitoring guidelines. The relative accessibility and autologous potential of MSC-derived mitochondria may simplify certain ethical considerations compared to embryonic stem cell-based approaches (Figure 3). However, rigorous evaluation of long-term immunologic and metabolic consequences will still be necessary prior to widespread clinical adoption.

In sum, while the ocular tissues are characterized by easy accessibility, the transition of mitochondrial transplantation from an experimental concept to a clinically viable therapy for ocular trauma will require overcoming substantial challenges. Despite these limitations, continued advances in mitochondrial preservation technologies, bioengineering strategies, and organelle tracking systems suggest that many of these barriers are addressable in the near future. As manufacturing protocols become increasingly standardized and our understanding deepens, mitochondrial transplantation using autologous source to sidestep immune rejection could evolve from an experimental rescue strategy into a reproducible platform for regenerative ophthalmology.

While proof-of-concept studies in corneal, retinal, and optic nerve models provide encouraging early data, mitochondrial transplantation remains a preclinical technology in ophthalmology. Current evidence supports mechanistic plausibility and therapeutic potential; however, rigorous studies addressing dosing, delivery routes, long-term mitochondrial engraftment, safety, and clinical efficacy are required before translation into routine ocular regenerative therapies.

## Figures and Tables

**Table 1:** Routes of Exogenous Mitochondrial Administration for Ocular Rescue.

Major Limitations	Primary Target Tissues	Current Evidence Level	Isolated Mitochondria Tested in Ocular Models?	Delivery Route
Uncertain mitochondrial stability in tear fluid; rapid clearance; mucin interaction; limited penetration beyond the epithelial barrier.	Corneal Epithelium, Limbal Niche	Preclinical Evidence	Yes	Topical/Eye Drops
Lack of direct evidence demonstrating organelle migration into internal ocular tissues; potential local inflammatory risk.	Anterior Segment, Corneal Stroma, Sclera	Conceptual/Extrapolated (no direct in vivo ocular data)	No	Subconjunctival Injection
Variable stability within vitreous humor; risk of inflammatory vitreous reaction; non-specific cell uptake; rapid clearance kinetics.	Retinal Ganglion Cells (RGCs), Inner Retina, Optic Nerve Head	Preclinical Evidence	Yes	Intravitreal Injection
Invasive surgical procedure; localized therapeutic effect restricted to the injection bleb area; risk of mechanical retinal detachment.	Outer Retina, Photoreceptors, Retinal Pigment Epithelium (RPE)	Preclinical Evidence (Limited)	Yes	Subretinal Injection

## Data Availability

The original contributions presented in the study are included in the article. Further inquiries can be directed to the corresponding authors.

## Abbreviations

Adenosine triphosphate (ATP), Electroretinogram (ERG), extracellular vesicles (EV), mesenchymal stem cells (MSCs), mitochondrial membrane potential (ΔΨm), mitochondrial permeability transition pore (mPTP), oxygen consumption rate (OCR), reactive oxygen species (ROS), traumatic optic neuropathy (TON), tunneling nanotubes (TNTs), retinal ischemia–reperfusion (*I/R*), retinal ganglion cells (RGC), urine-derived stem cells (USC).

## References

[1] Felizarta M.J.A., Soleimani M. (2026). Editorial: Epidemiological characteristics of ocular trauma globally and their clinical implications. Front. Med..

[2] Kuhn F., Morris R., Witherspoon C.D., Heimann K., Jeffers J.B., Treister G. (1996). A standardized classification of ocular trauma. Ophthalmology.

[3] Eslani M., Baradaran-Rafii A., Movahedan A., Djalilian A.R. (2014). The ocular surface chemical burns. J. Ophthalmol..

[4] Patek G.C., Bates A., Gurnani B. (2026). Ocular Burns. StatPearls.

[5] Ayanniyi A.A., Fasasi M.K. (2013). Uniocular Blindness Following Thermal Injury. Malays. J. Med. Sci..

[6] Verma S., Waikar S., Sharma V., Bhatkoti B., Chauhan R. (2021). Ocular trauma in counter insurgency and proxy war environment: Epidemiological study, 1992–2004. Med. J. Armed Forces India.

[7] Liu D., Zhang C., Zhang J., Xu G.-T., Zhang J. (2023). Molecular pathogenesis of subretinal fibrosis in neovascular AMD focusing on epithelial-mesenchymal transformation of retinal pigment epithelium. Neurobiol. Dis..

[8] Zhu J., Li P., Zhou Y.-G., Ye J. (2020). Altered Energy Metabolism During Early Optic Nerve Crush Injury: Implications of Warburg-Like Aerobic Glycolysis in Facilitating Retinal Ganglion Cell Survival. Neurosci. Bull..

[9] Chen Y., Coorey N.J., Zhang M., Zeng S., Madigan M.C., Zhang X., Gillies M.C., Zhu L., Zhang T. (2022). Metabolism Dysregulation in Retinal Diseases and Related Therapies. Antioxidants.

[10] Kalogeris T., Baines C.P., Krenz M., Korthuis R.J. (2012). Cell Biology of Ischemia/Reperfusion Injury. Int. Rev. Cell Mol. Biol..

[11] Lemasters J.J., Theruvath T.P., Zhong Z., Nieminen A.-L. (2009). Mitochondrial Calcium and the Permeability Transition in Cell Death. Biochim. Biophys. Acta.

[12] Gupta S., Kass G.E., Szegezdi E., Joseph B. (2009). The mitochondrial death pathway: A promising therapeutic target in diseases. J. Cell. Mol. Med..

[13] Fung A.T., Tran T., Lim L.L., Samarawickrama C., Arnold J., Gillies M., Catt C., Mitchell L., Symons A., Buttery R. (2020). Local delivery of corticosteroids in clinical ophthalmology: A review. Clin. Exp. Ophthalmol..

[14] Siqueira R.C., Brandão C.C. (2025). The Role of Cytokines in Degenerative Retinal Diseases: A Comprehensive Review. Biomedicines.

[15] Feroze K.B., Zeppieri M., Khazaeni L. (2026). Steroid-Induced Glaucoma. StatPearls.

[16] Saccu G., Menchise V., Giordano C., Castelli D.D., Dastrù W., Pellicano R., Tolosano E., Van Pham P., Altruda F., Fagoonee S. (2021). Regenerative Approaches and Future Trends for the Treatment of Corneal Burn Injuries. J. Clin. Med..

[17] Giorgi C., Marchi S., Pinton P. (2018). The machineries, regulation and cellular functions of mitochondrial calcium. Nat. Rev. Mol. Cell Biol..

[18] Osborne N.N., Casson R.J., Wood J.P., Chidlow G., Graham M., Melena J. (2004). Retinal ischemia: Mechanisms of damage and potential therapeutic strategies. Prog. Retin. Eye Res..

[19] Bernardi P., Gerle C., Halestrap A.P., Jonas E.A., Karch J., Mnatsakanyan N., Pavlov E., Sheu S.-S., Soukas A.A. (2023). Identity, structure, and function of the mitochondrial permeability transition pore: Controversies, consensus, recent advances, and future directions. Cell Death Differ..

[20] Knöferle J., Koch J.C., Ostendorf T., Michel U., Planchamp V., Vutova P., Tönges L., Stadelmann C., Brück W., Bähr M. (2010). Mechanisms of acute axonal degeneration in the optic nerve in vivo. Proc. Natl. Acad. Sci. USA.

[21] Crish S.D., Calkins D.J. (2011). Neurodegeneration in glaucoma: Progression and calcium-dependent intracellular mechanisms. Neuroscience.

[22] Kitani T., Kami D., Matoba S., Gojo S. (2014). Internalization of isolated functional mitochondria: Involvement of macropinocytosis. J. Cell. Mol. Med..

[23] Zamberlan M., Semenzato M. (2025). Mechanisms of Mitochondrial Transfer Through TNTs: From Organelle Dynamics to Cellular Crosstalk. Int. J. Mol. Sci..

[24] Yang J., Liu L., Oda Y., Wada K., Ago M., Matsuda S., Hattori M., Goto T., Ishibashi S., Kawashima-Sonoyama Y. (2023). Extracellular Vesicles and Cx43-Gap Junction Channels Are the Main Routes for Mitochondrial Transfer from Ultra-Purified Mesenchymal Stem Cells, RECs. Int. J. Mol. Sci..

[25] Miller M.A., Zachary J.F. (2017). Mechanisms and Morphology of Cellular Injury, Adaptation, and Death. Pathol. Basis Vet. Dis..

[26] Khalaji A., Yancheshmeh F.B., Farham F., Khorram A., Sheshbolouki S., Zokaei M., Vatankhah F., Soleymani-Goloujeh M. (2023). Don’t eat me/eat me signals as a novel strategy in cancer immunotherapy. Heliyon.

[27] Alberts B., Johnson A., Lewis J., Raff M., Roberts K., Walter P. (2002). The Mitochondrion. Molecular Biology of the Cell.

[28] Patel D., Rorbach J., Downes K., Szukszto M.J., Pekalski M.L., Minczuk M. (2017). Macropinocytic entry of isolated mitochondria in epidermal growth factor-activated human osteosarcoma cells. Sci. Rep..

[29] Gökerküçük E.B., Tramier M., Bertolin G. (2020). Imaging mitochondrial functions: From fluorescent dyes to genetically-encoded sensors. Genes.

[30] Chen H., Yu Z., Ren S., Qiu Y. (2022). Fluorescent probes design strategies for imaging mitochondria and lysosomes. Front. Pharmacol..

[31] Kubat G.B., Picone P., Tuncay E., Aryan L., Girgenti A., Palumbo L., Turkel I., Akat F., Singh K.K., Nuzzo D. (2025). Biotechnological approaches and therapeutic potential of mitochondria transfer and transplantation. Nat. Commun..

[32] Naji A., Eitoku M., Favier B., Deschaseaux F., Rouas-Freiss N., Suganuma N. (2019). Biological functions of mesenchymal stem cells and clinical implications. Cell. Mol. Life Sci..

[33] Boysen A.T., Whitehead B., Revenfeld A.L.S., Gupta D., Petersen T., Nejsum P. (2024). Urine-derived stem cells serve as a robust platform for generating native or engineered extracellular vesicles. Stem Cell Res. Ther..

[34] Velarde F., Ezquerra S., Delbruyere X., Caicedo A., Hidalgo Y., Khoury M. (2022). Mesenchymal stem cell-mediated transfer of mitochondria: Mechanisms and functional impact. Cell. Mol. Life Sci..

[35] Zhang D., Wei G., Li P., Zhou X., Zhang Y. (2014). Urine-derived stem cells: A novel and versatile progenitor source for cell-based therapy and regenerative medicine. Genes Dis..

[36] Mohseni M., Gurnani B., Blair K. (2026). Blunt Eye Trauma. StatPearls.

[37] Rastegar F., Shenaq D., Huang J., Zhang W., Zhang B.-Q., He B.-C., Chen L., Zuo G.-W., Luo Q., Shi Q. (2010). Mesenchymal stem cells: Molecular characteristics and clinical applications. World J. Stem Cells.

[38] Phinney D.G., Di Giuseppe M., Njah J., Sala E., Shiva S., St Croix C.M., Stolz D.B., Watkins S.C., Di Y.P., Leikauf G.D. (2015). Mesenchymal stem cells use extracellular vesicles to outsource mitophagy and shuttle microRNAs. Nat. Commun..

[39] Spees J.L., Olson S.D., Whitney M.J., Prockop D.J. (2006). Mitochondrial transfer between cells can rescue aerobic respiration. Proc. Natl. Acad. Sci. USA.

[40] Chen H., Chen X., Zhou Z.-H., Zheng J.-R., Lu Y., Lin P., Lin Y.-F., Zheng Y.-C., Xiong B., Xu R.-W. (2025). Mesenchymal stromal cell-mediated mitochondrial transfer unveils new frontiers in disease therapy. Stem Cell Res. Ther..

[41] Jiang D., Yu J., Wu X., Yu X., Jin P., Zheng H., Liu H., Xu W., Lian Q., Chen W. (2025). Isolated Mitochondrial Transplantation as a Novel Treatment for Corneal Chemical Burns. Investig. Ophthalmol. Vis. Sci..

[42] McCully J.D., Levitsky S., Del Nido P.J., Cowan D.B. (2016). Mitochondrial transplantation for therapeutic use. Clin. Transl. Med..

[43] Giuffrida E., Plantania C., Lazzara F., Conti F., Sotera L., Drago F., Herath D., Motterlini R., Foresti R., Bucolo C. (2025). α1D adrenergic receptor antagonism protects against high glucose-induced mitochondrial dysfunction and blood retinal barrier breakdown in ARPE-19 cells. Int. J. Mol. Sci..

[44] Pittalà V., Salerno L., Romeo G., Siracusa M.A., Modica M.N., Romano G.L., Salomone S., Drago F., Bucolo C. (2015). Effects of novel hybrids of caffeic acid phenethyl ester and NSAIDs on experimental ocular inflammation. Eur. J. Pharmacol..

[45] Khan R.S., Ross A.G., Aravand P., Dine K., Selzer E.B., Shindler K.S. (2021). RGC and Vision Loss From Traumatic Optic Neuropathy Induced by Repetitive Closed Head Trauma Is Dependent on Timing and Force of Impact. Transl. Vis. Sci. Technol..

[46] Liu H., Prokosch V. (2021). Energy Metabolism in the Inner Retina in Health and Glaucoma. Int. J. Mol. Sci..

[47] Nascimento-dos-Santos G., De-Souza-Ferreira E., Lani R., Faria C.C., Araújo V.G., Teixeira-Pinheiro L.C., Vasconcelos T., Gonçalo T., Santiago M.F., Linden R. (2020). Neuroprotection from optic nerve injury and modulation of oxidative metabolism by transplantation of active mitochondria to the retina. Biochim. Biophys. Acta Mol. Basis Dis..

[48] Hartsock M.J., Cho H., Wu L., Chen W.-J., Gong J., Duh E.J. (2016). A Mouse Model of Retinal Ischemia-Reperfusion Injury Through Elevation of Intraocular Pressure. J. Vis. Exp..

[49] Liang X., Zhang Y., Lin F., Li M., Li X., Chen Y., Liu J., Meng Q., Ma X., Wang E. (2022). Direct administration of mesenchymal stem cell-derived mitochondria improves cardiac function after infarction via ameliorating endothelial senescence. Bioeng. Transl. Med..

[50] Hayakawa K., Esposito E., Wang X., Terasaki Y., Liu Y., Xing C., Ji X., Lo E.H. (2016). Transfer of mitochondria from astrocytes to neurons after stroke. Nature.

[51] Lin M.-W., Fang S.-Y., Hsu J.-Y.C., Huang C.-Y., Lee P.-H., Huang C.-C., Chen H.-F., Lam C.-F., Lee J.-S. (2022). Mitochondrial transplantation attenuates neural damage and improves locomotor function after traumatic spinal cord injury in rats. Front. Neurosci..

[52] Islam M.N., Das S.R., Emin M.T., Wei M., Sun L., Westphalen K., Rowlands D.J., Quadri S.K., Bhattacharya S., Bhattacharya J. (2012). Mitochondrial transfer from bone-marrow-derived stromal cells to pulmonary alveoli protects against acute lung injury. Nat. Med..

[53] Dubinin M.V., Mikheeva I.B., Stepanova A.E., Igoshkina A.D., Cherepanova A.A., Semenova A.A., Sharapov V.A., Kireev I.I., Belosludtsev K.N. (2024). Mitochondrial transplantation therapy ameliorates muscular dystrophy in mdx mouse model. Biomolecules.

[54] Calonge M., Nieto-Miguel T., de la Mata A., Galindo S., Herreras J.M., López-Paniagua M. (2021). Goals and Challenges of Stem Cell-Based Therapy for Corneal Blindness Due to Limbal Deficiency. Pharmaceutics.

[55] Soleimani M., Masoumi A., Momenaei B., Cheraqpour K., Koganti R., Chang A.Y., Ghassemi M., Djalilian A.R. (2023). Applications of mesenchymal stem cells in ocular surface diseases: Sources and routes of delivery. Expert Opin. Biol. Ther..

[56] Casini G., Sartini F., Loiudice P., Benini G., Menchini M. (2021). Ocular siderosis: A misdiagnosed cause of visual loss due to ferrous intraocular foreign bodies-epidemiology, pathogenesis, clinical signs, imaging and available treatment options. Doc. Ophthalmol..

[57] Mattei V., Santilli F., Pulcini F., Fabrizi J., Lancia L., Santacroce C., Megiorni F., Ceccarelli S., Paldino E., Gramignoli R. (2025). Validated methods for isolation and qualification of mesenchymal stromal/stem cells from different sources. J. Transl. Med..

[58] Martini H., Passos J.F. (2023). Cellular senescence: All roads lead to mitochondria. FEBS J..

[59] Horcharoensuk P., Yang-en S., Narkwichean A., Rungsiwiwut R. (2022). Proline-based solution maintains cell viability and stemness of canine adipose-derived mesenchymal stem cells after hypothermic storage. PLoS ONE.

[60] Okutani A., Naima J., Ogihara A., Motoichi T., Ohsawa I., Ohta Y. (2025). A method for isolating and cryopreserving intact mitochondria with improved integrity and functionality. Biophys. Physicobiology.

[61] Knight L.J., Martis R.M., Donaldson P.J., Acosta M.L., Lim J.C. (2025). Changes in Redox Balance and Mitochondrial Activity in the Retinas of Cystine/Glutamate Antiporter Knockout Mice. Investig. Ophthalmol. Vis. Sci..

[62] Yeo Y.Z., Bhargava M., Khare R.S., Parikh B.H., Su X. (2025). Mitochondrial transplant: Unravelling a promising treatment for ocular diseases. Redox Biol..

[63] Perciballi E., Bovio F., Ferro S., Forcella M., Rosati J., Carletti R.M., D’aNzi A., Gelati M., La Bella V., Innocenti M. (2025). Mitochondrial and energy metabolism dysfunctions are hallmarks of TDP-43G376D fibroblasts from members of an Amyotrophic Lateral Sclerosis family. Cell Death Dis..

[64] Sabirova S., Sharapova G., Budyukova A., Gomzikova M., Brichkina A., Barlev N.A., Rizvanov A., Markov N., Simon H.-U. (2025). Comprehensive analysis of cellular metrics: From proliferation to mitochondrial membrane potential and cell death in a single sample. Cell Death Dis..

[65] Baig J., Pradeepkiran J.A., Reddy P.H. (2023). Methods to Study the Mitochondria—qRT-PCR, Western Blotting, Immunofluorescence, Electron Microscopy, “Fluorescence”, and Seahorse—Techniques used to study the effects of age-related diseases like Alzheimer’s. Curr. Protoc..

[66] Tan F., Li X., Wang Z., Li J., Shahzad K., Zheng J. (2024). Clinical applications of stem cell-derived exosomes. Signal Transduct. Target. Ther..

[67] Samaeekia R., Rabiee B., Putra I., Shen X., Park Y.J., Hematti P., Eslani M., Djalilian A.R. (2018). Effect of human corneal mesenchymal stromal cell-derived exosomes on corneal epithelial wound healing. Investig. Ophthalmol. Vis. Sci..

[68] Saeedi P., Nilchiani L.S., Zand B., Hajimirghasemi M., Halabian R. (2025). An overview of stem cells and cell products involved in trauma injury. Regen. Ther..

[69] Movahed A.Y., Bagheri R., Savatier P., Šarić T., Moradi S. (2025). Elimination of tumorigenic pluripotent stem cells from their differentiated cell therapy products: An important step toward ensuring safe cell therapy. Stem Cell Rep..

[70] Chen S., Zhang L., Ren Y., Bai X., Wei X., Chai M., Cui S., Zhao X., Deng H. (2026). Clinical translational research on stem cell products: Prospects and challenges. Signal Transduct. Target. Ther..

[71] Yi Y.-F., Fan Z.-Q., Liu C., Ding Y.-T., Chen Y., Wen J., Jian X.-H., Li Y.-F. (2025). Immunomodulatory effects and clinical application of exosomes derived from mesenchymal stem cells. World J. Stem Cells.

[72] Wu K.Y., Ahmad H., Lin G., Carbonneau M., Tran S.D. (2023). Mesenchymal Stem Cell-Derived Exosomes in Ophthalmology: A Comprehensive Review. Pharmaceutics.

[73] Hu L., Duan H., Zhang Y., Yang L., Yoon K.C., Shen Z., Li Z., Ma B., Qi H. (2026). The Therapeutic Potential of Exosomes in Ocular Surface Diseases. Biomolecules.

[74] Taghavi-Farahabadi M., Mahmoudi M., Soudi S., Hashemi S.M. (2020). Hypothesis for the management and treatment of the COVID-19-induced acute respiratory distress syndrome and lung injury using mesenchymal stem cell-derived exosomes. Med. Hypotheses.

[75] Ranjan P., Colin K., Dutta R.K., Verma S.K. (2023). Challenges and future scope of exosomes in the treatment of cardiovascular diseases. J. Physiol..

[76] Miao X., Jiang P., Wang Z., Kong W., Feng L. (2025). Mitochondrial Transplantation: A Novel Therapeutic Approach for Treating Diseases. MedComm.

[77] Zhang M., Wu J., Cai K., Liu Y., Lu B., Zhang J., Xu J., Gu C., Chen T. (2024). From dysfunction to healing: Advances in mitochondrial therapy for Osteoarthritis. J. Transl. Med..

[78] Picard M., Taivassalo T., Gouspillou G., Hepple R.T. (2011). Mitochondria: Isolation, structure and function. J. Physiol..

[79] Mohamed-Ahmed S., Fristad I., Lie S.A., Suliman S., Mustafa K., Vindenes H., Idris S.B. (2018). Adipose-derived and bone marrow mesenchymal stem cells: A donor-matched comparison. Stem Cell Res. Ther..

[80] Allouh M.Z., Rizvi S.F.A., Alamri A., Jimoh Y., Aouda S., Ouda Z.H., Hamad M.I.K., Perez-Cruet M., Chaudhry G.R. (2025). Mesenchymal stromal/stem cells from perinatal sources: Biological facts, molecular biomarkers, and therapeutic promises. Stem Cell Res. Ther..

[81] Yu P., Bosholm C.C., Zhu H., Duan Z., Atala A., Zhang Y. (2024). Beyond waste: Understanding urine’s potential in precision medicine. Trends Biotechnol..

[82] Baer P.C., Geiger H. (2012). Adipose-Derived Mesenchymal Stromal/Stem Cells: Tissue Localization, Characterization, and Heterogeneity. Stem Cells Int..

[83] Wang J., Li R., Qian L. (2026). Intercellular mitochondrial transfer rewires redox signaling and metabolic plasticity: Mechanisms, disease relevance and therapeutic frontiers. Redox Biol..

[84] Rodríguez-Nuevo A., Zorzano A. (2019). The sensing of mitochondrial DAMPs by non-immune cells. Cell Stress.

